# Isolated bursal-side infraspinatus tear diagnosed by computed tomography bursography: a case series

**DOI:** 10.1186/s13256-022-03472-x

**Published:** 2022-06-17

**Authors:** Yoshihiro Onada, Hideyuki Sasanuma, Takahisa Umemoto, Tomomichi Kajino, Tadashi Shimizu

**Affiliations:** 1 Department of Orthopaedic Surgery, Tonan Hospital, 3-8 Kita-4-jo Nishi-7-chome, Chuo-ku, Sapporo, Hokkaido 060-0004 Japan; 2 Department of Sports Health Medicine, Tochigi Medical Center Shimotsuga, Tochigi, Japan; 3 Department of Radiology, Tonan Hospital, Sapporo, Hokkaido Japan

**Keywords:** Bursography, Magnetic resonance imaging, Partial-thickness rotator cuff tear, Infraspinatus, Shoulder impingement syndrome

## Abstract

**Background:**

Partial-thickness rotator cuff tears are commonly found in the articular-side tendon of the supraspinatus; however, isolated lesions, except those occurring in the supraspinatus tendons, are rare. We report three cases of isolated bursal-side infraspinatus tears that were difficult to detect by magnetic resonance imaging but could be visualized by computed tomography bursography.

**Case presentation:**

Three Asian patients (59–71 years old) with shoulder pain ranging from 1 month to 3 years in duration were each diagnosed with shoulder impingement syndrome. Magnetic resonance imaging studies failed to show a tear of the rotator cuff tendon complex. However, computed tomography bursography showed a longitudinal infraspinatus partial-thickness tear on the bursal side in each case. Arthroscopic decompression of the subacromial space and debridement of the infraspinatus tendon tear successfully alleviated the shoulder pain in two of the three patients; the third patient did not undergo surgery and remained symptomatic at follow-up.

**Conclusions:**

In patients with chronic shoulder pain but normal magnetic resonance imaging findings, computed tomography bursography should be considered, as bursal-side infraspinatus tears may be found in these patients. Furthermore, our findings provide a basis for larger studies to further establish the accuracy of computed tomography bursography for these lesions.

## Background

Partial-thickness rotator cuff tears (PTRCTs) are a common cause of adult shoulder pain, with a relatively high prevalence ranging from 13% to 32% [1]. However, diagnosis and treatment remain controversial. Based on cadaver and clinical studies, most PTRCTs involve the supraspinatus tendon, and articular-side tears are two to three times more common than bursal-side tears [2–5]. Isolated PTRCTs are rare, except those occurring in supraspinatus tendons [3]. Herein, we report three cases of isolated bursal-side infraspinatus tears that were difficult to detect by magnetic resonance imaging (MRI). In these cases, computed tomography (CT) bursography helped identify the lesion.

## Case presentation

### Case 1

**Fig. 1:**
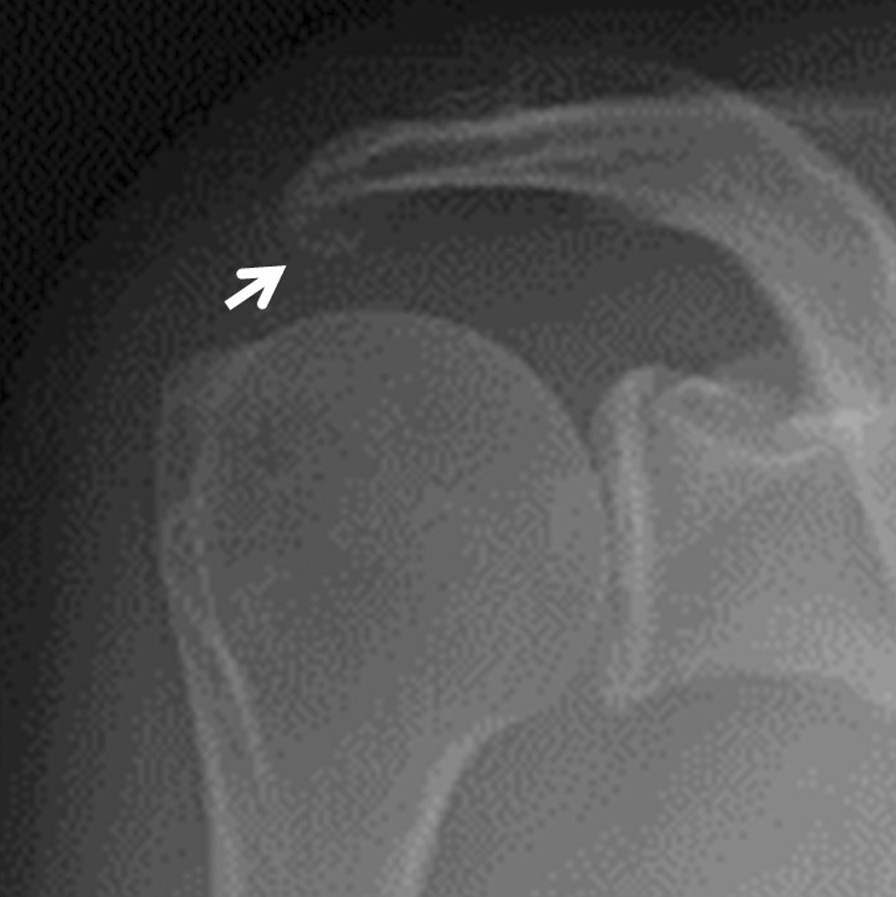
Anteroposterior radiograph of the right shoulder of a 59-year-old Asian woman showing a subacromial spur (arrow)

**Fig. 2:**
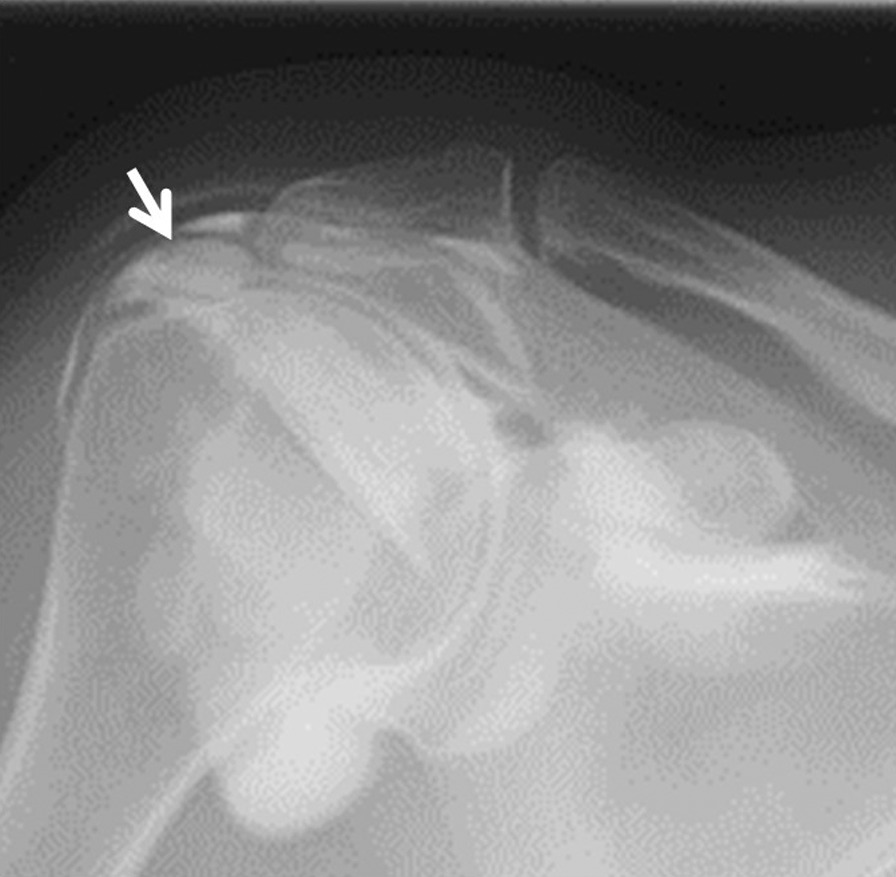
A 59-year-old Asian woman with an isolated bursal-side infraspinatus tear. Subacromial bursography shows localized pooling of contrast medium in a tendon area of the rotator cuff (arrow)

**Fig. 3:**
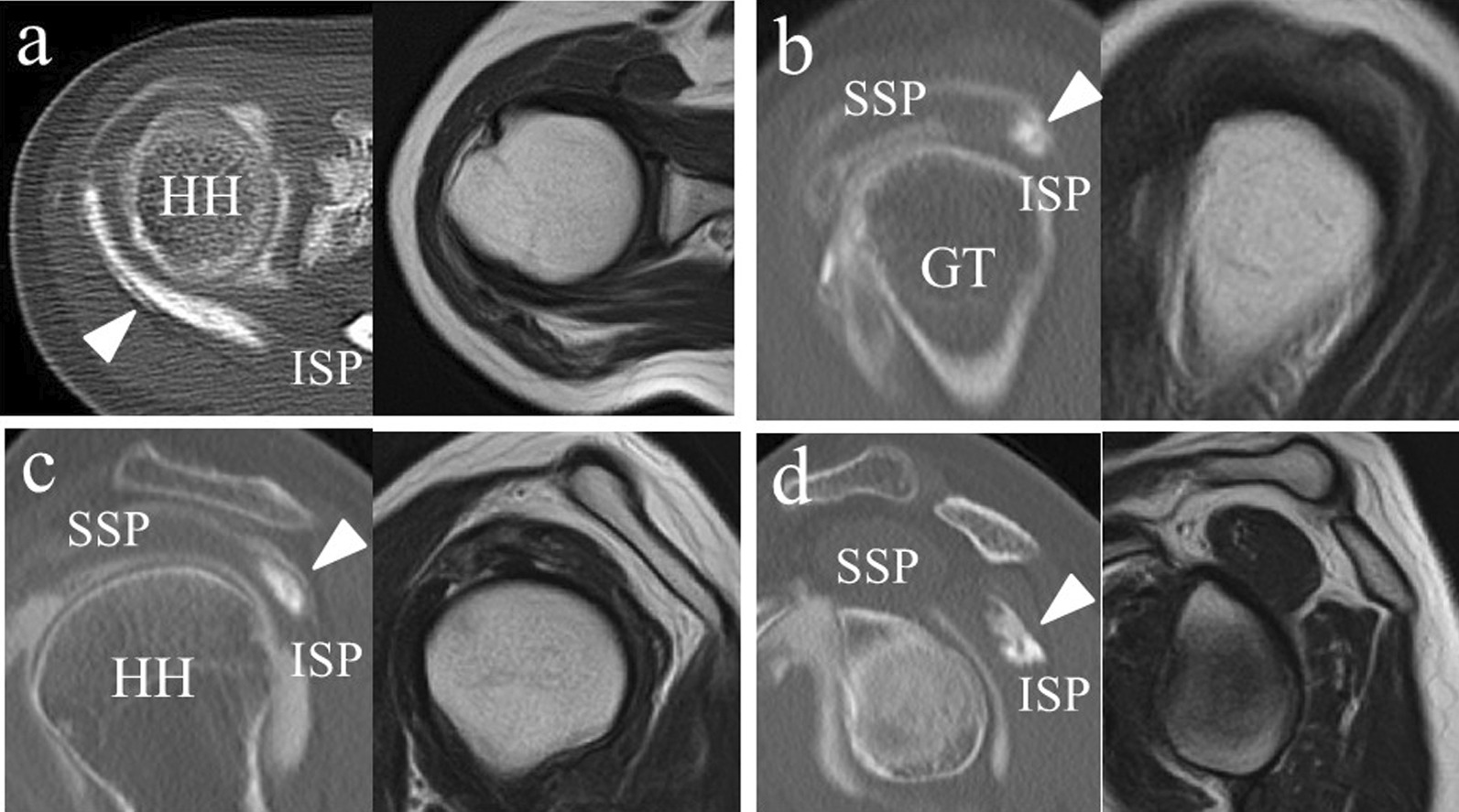
The infraspinatus tear is visualized by Computed tomography bursography. However, axial and sagittal T2-weighted MR images without fat suppression show a normal rotator cuff. **a** The axial image on Computed tomography bursography shows longitudinal pooling of contrast medium in the infraspinatus (arrowhead). **b** The sagittal image reveals that it communicates with the subacromial bursa near the insertion of the infraspinatus tendon (arrowhead) and (**c**, **d**) within the infraspinatus tendon and muscle body (arrowhead). *HH* head of the humerus, *GT* greater tubercle, *SSP* supraspinatus, *ISP* infraspinatus

**Fig. 4:**
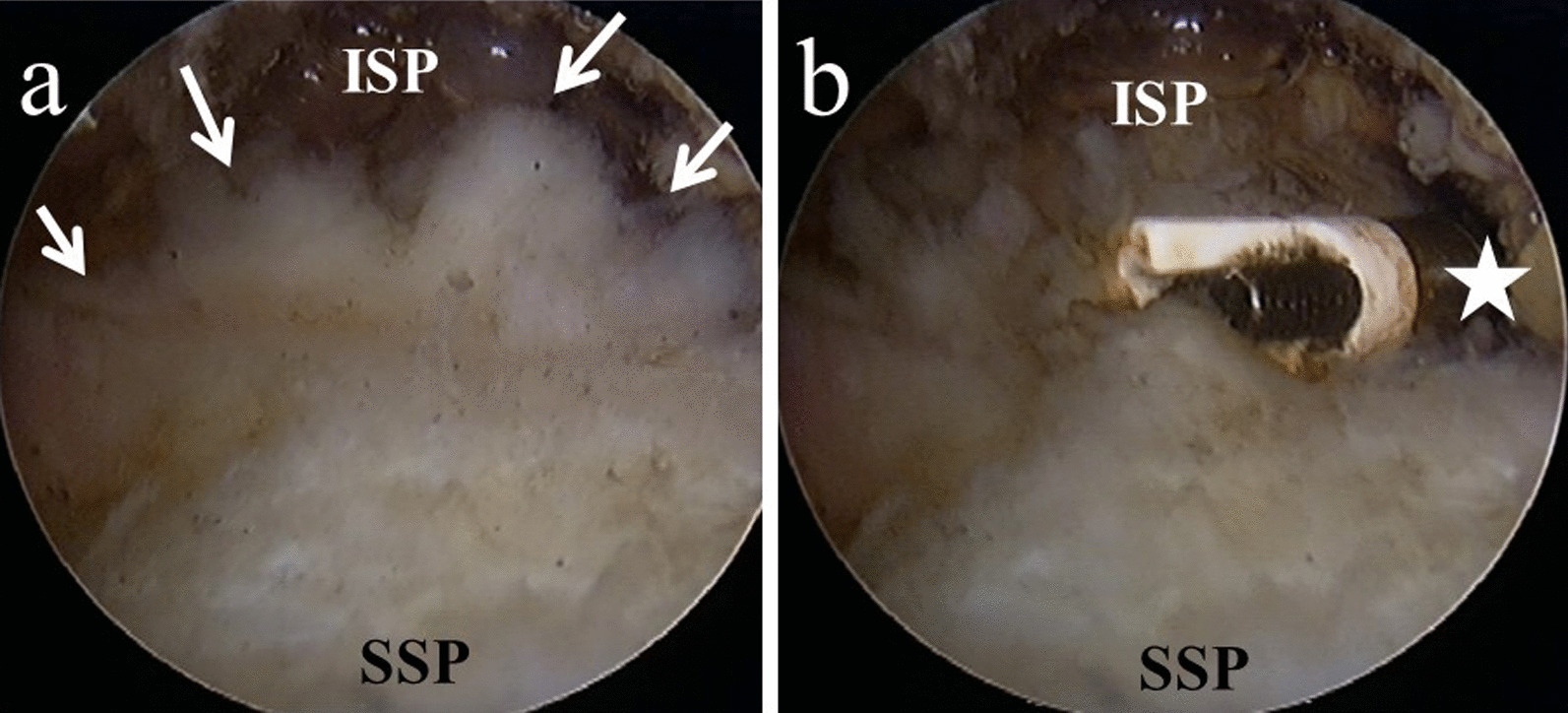
**a** Arthroscopy confirms the rotator cuff tear with a torn flap (arrows). **b** The flap is trimmed by a radiofrequency device (white star). *SSP* supraspinatus, *ISP* infraspinatus

A 59-year-old Asian woman with no previous history of trauma presented with 2-year history of severe shoulder pain. She underwent conservative management with steroidal injection and physiotherapy for at least 3 months at the referral hospital. On physical examination at the first visit to our institute, there was tenderness over the greater tubercle, and Neer’s test and Hawkins–Kennedy test elicited pain. Radiographs showed a subacromial spur (Fig. 1), and T2-weighted MRI with field strength of 1.5 T demonstrated a normal rotator cuff. Based on these findings, shoulder impingement syndrome was diagnosed, and the patient was managed conservatively with combinations of nonsteroid antiinflammatory medication, a steroid injection into the subacromial space, and physical therapy. However, no improvement was noted after 1 month, and the patient reported her shoulder pain as intolerable. Arthrography was performed using contrast mixed with lidocaine. The image showed no leakage, and the lidocaine did not alleviate the shoulder pain. Therefore, bursography was performed, and the image showed pooling of the contrast medium in a tendon area of the rotator cuff (Fig. 2). In addition, the shoulder pain was relieved. CT bursography clearly revealed a longitudinal infraspinatus tear (Fig. 3a–d). This lesion could not be identified on the MRI obtained 1 month earlier (Fig. 3a–d). Subsequent arthroscopy identified the bursal-side infraspinatus tear (Fig. 4a), and the torn flap was trimmed after subacromial decompression (Fig. 4b). At 2 years postoperatively, the patient remained free of shoulder pain.

### Case 2

**Fig. 5:**
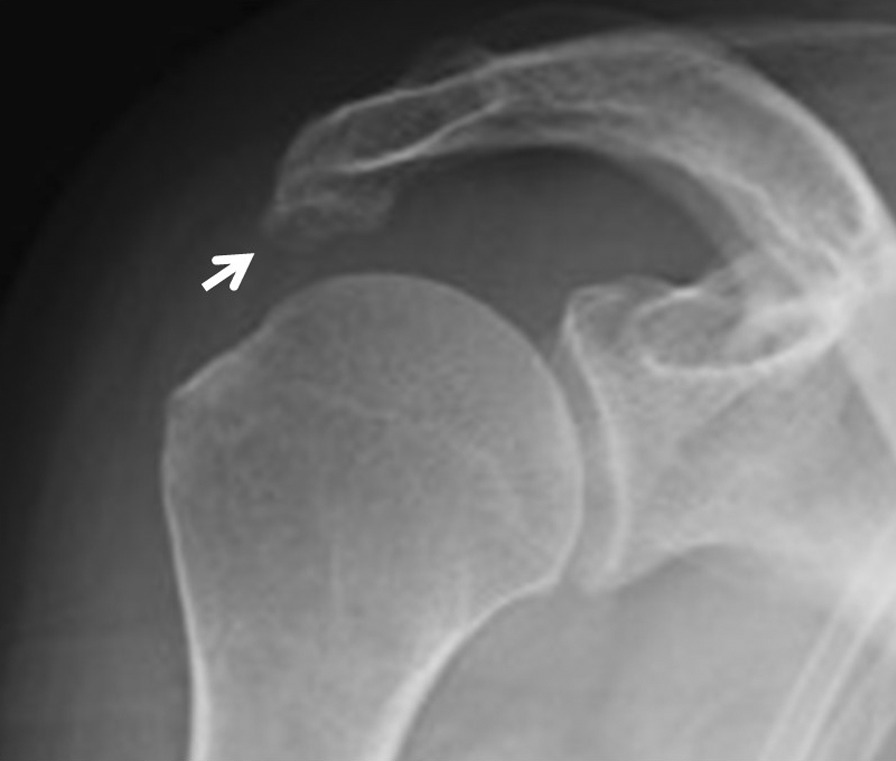
Anteroposterior radiograph of the right shoulder of a 59-year-old Asian woman showing a marked subacromial spur (arrow)

**Fig. 6:**
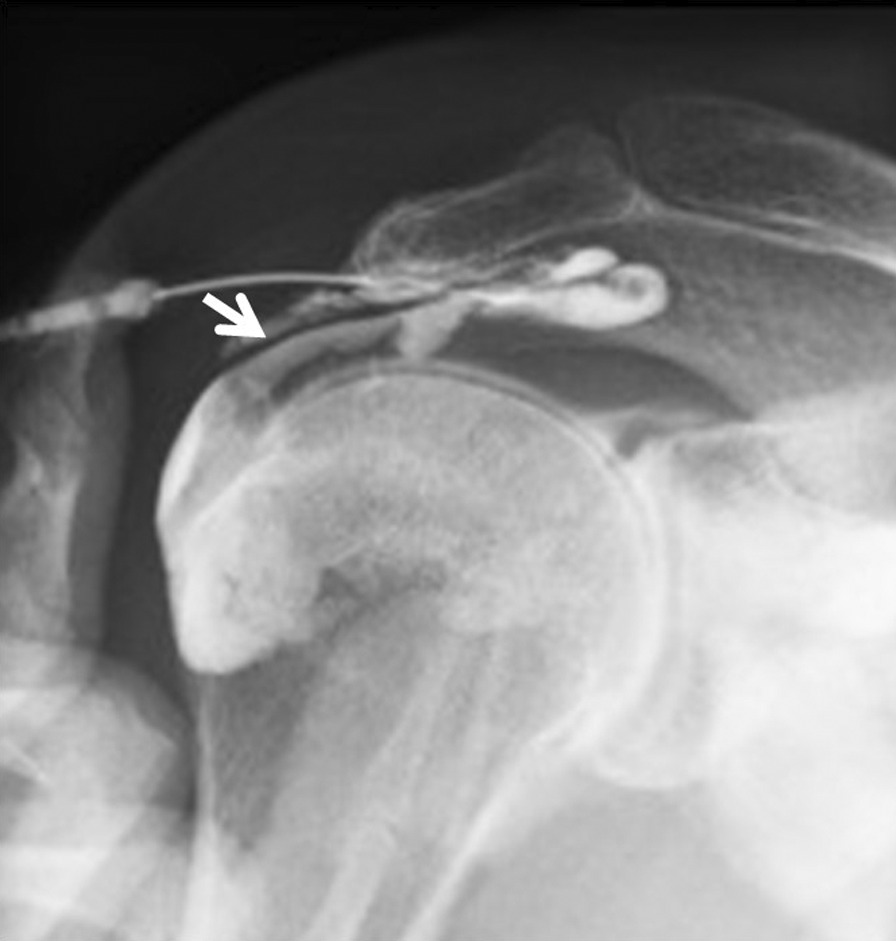
A 59-year-old Asian woman with isolated bursal-side infraspinatus tear. Subacromial bursography shows pooling of contrast medium in a tendon area of the rotator cuff (arrow)

**Fig. 7:**
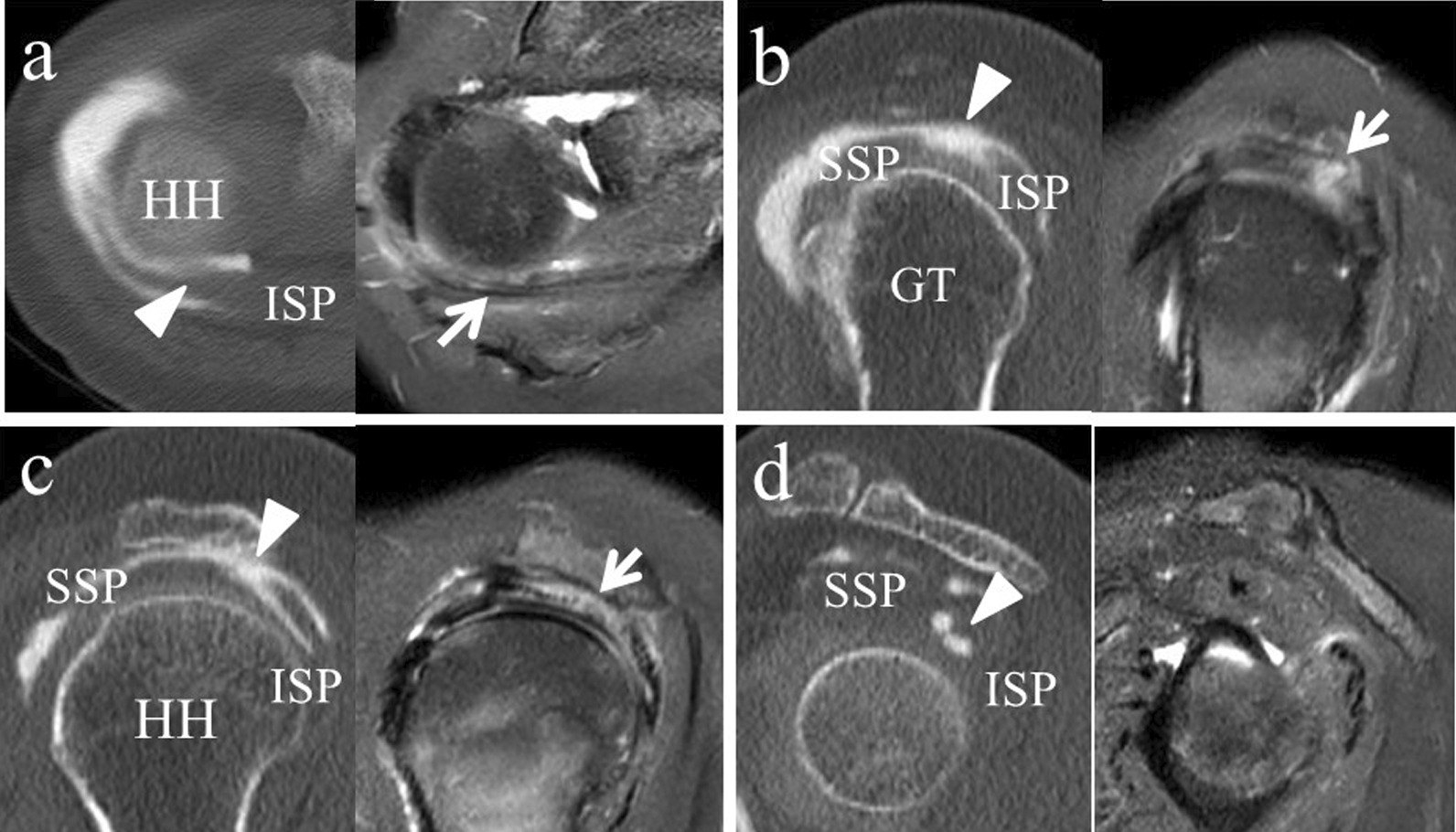
The tear is clearly visualized by Computed tomography bursography. On fat-suppressed T2-weighted Magnetic resonance imaging, there appears to be increased signal in the infraspinatus, suggesting a bursal-side tear. **a** Axial image shows longitudinal pooling of contrast medium in the infraspinatus (arrowhead) on Computed tomography bursography. The Magnetic resonance imaging shows areas of an increased signal in the infraspinatus (arrows). On Computed tomography bursography (**b**, **c**), the oblique sagittal image reveals that the tear communicates with the subacromial bursa in the infraspinatus tendon (arrowhead). The Magnetic resonance imaging shows areas of increased signal in the infraspinatus tendon, which is in contact with the bursal side of the rotator cuff (arrows). **d** Computed tomography bursography shows pooling of contrast medium within the muscle (arrowhead). *HH* head of the humerus, *GT* greater tubercle, *SSP* supraspinatus, *ISP* infraspinatus

**Fig. 8:**
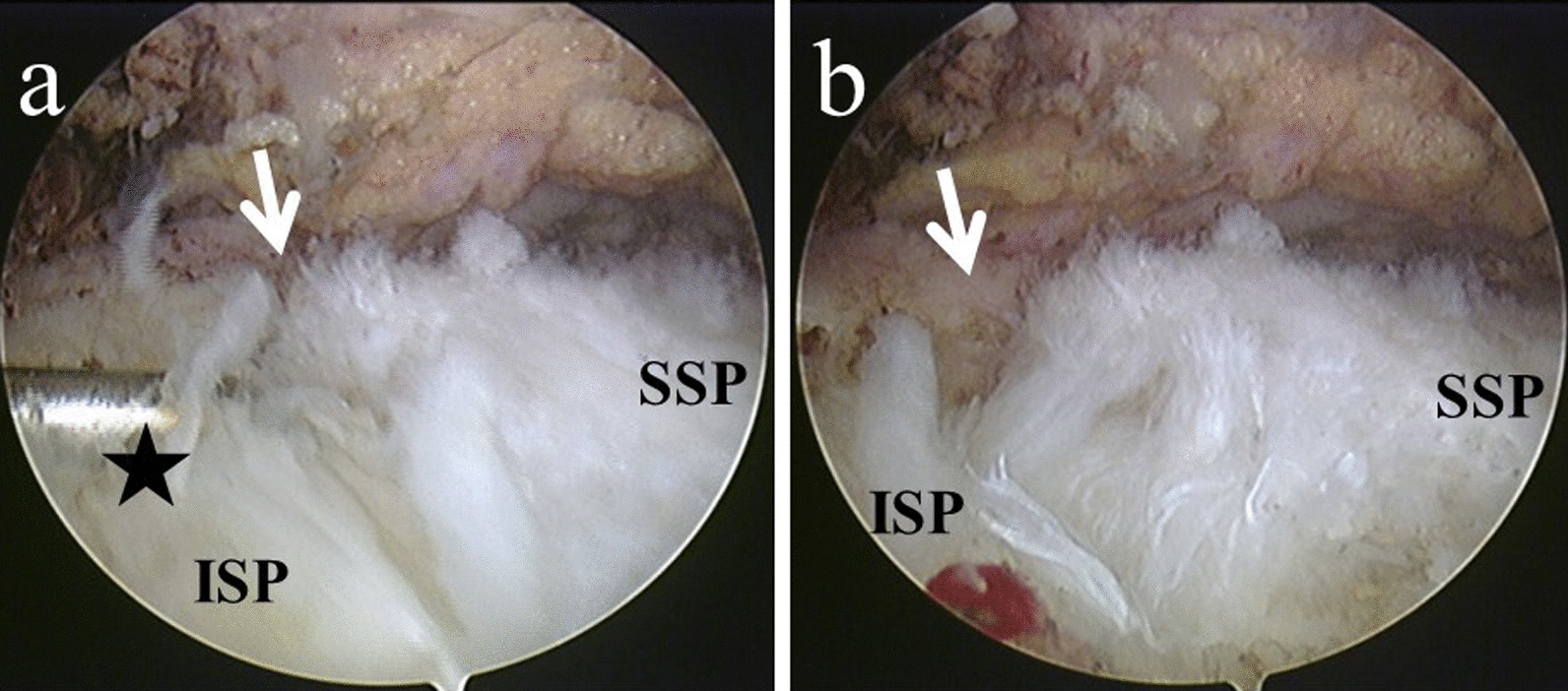
**a** Arthroscopy confirmed the rotator cuff tear with a torn flap (arrow), and the tear was examined with a hook-shaped probe (black star). **b** The rotator cuff tear gaped (arrow) when the torn flap was pulled posteriorly by the probe. *SSP* supraspinatus, *ISP* infraspinatus

A 59-year-old Asian woman presented with 1-month history of shoulder pain. She did not report any history of trauma. Examination revealed tenderness over the greater tubercle, and pain was elicited on performing Neer’s test. A marked subacromial spur could be seen on radiographs (Fig. 5), and a T2-weighted MRI with field strength of 1.5 T demonstrated a normal rotator cuff. Accordingly, shoulder impingement syndrome was diagnosed, and she was treated conservatively for 6 months. However, symptoms did not improve with conservative treatment, and the patient requested surgical treatment. Her preoperative arthrogram with contrast and lidocaine showed no findings of note and did not relieve her shoulder pain. Hence, bursography was performed, showing pooling of the contrast medium in a tendon area of the rotator cuff (Fig. 6), and the patient reported alleviation of the shoulder pain. CT bursography revealed a longitudinal infraspinatus tear (Fig. 7a–d). Fat-suppressed T2-weighted MRI with a field strength of 1.5 T was performed within 1 month after the CT. Although the MRI images suggested a bursal-side PTRCT, the longitudinal lesion was difficult to identify (Fig. 7a–d). The patient underwent arthroscopy, which confirmed the bursal-side infraspinatus tear (Fig. 8a, b). The torn flap was trimmed after subacromial decompression. The patient’s shoulder remained pain-free 1 year later.

### Case 3

**Fig. 9:**
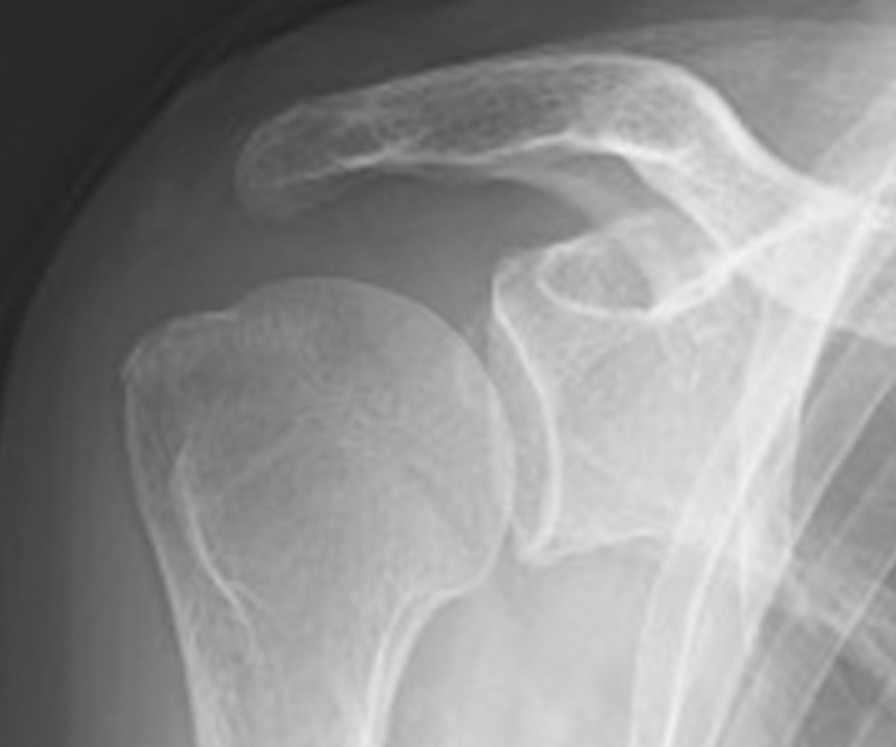
Anteroposterior radiograph of the right shoulder of a 71-year-old Asian woman. There is no apparent subacromial spur

**Fig. 10:**
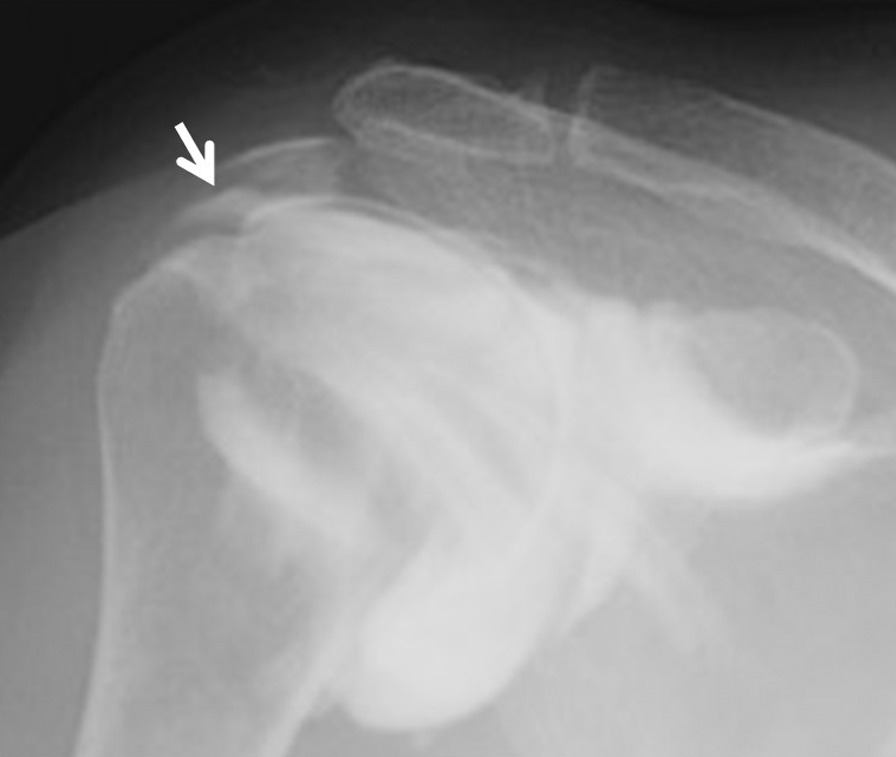
A 71-year-old Asian woman with an isolated bursal-side infraspinatus tear. Subacromial bursography shows localized pooling of contrast medium in a tendon area of the rotator cuff (arrow)

**Fig. 11:**
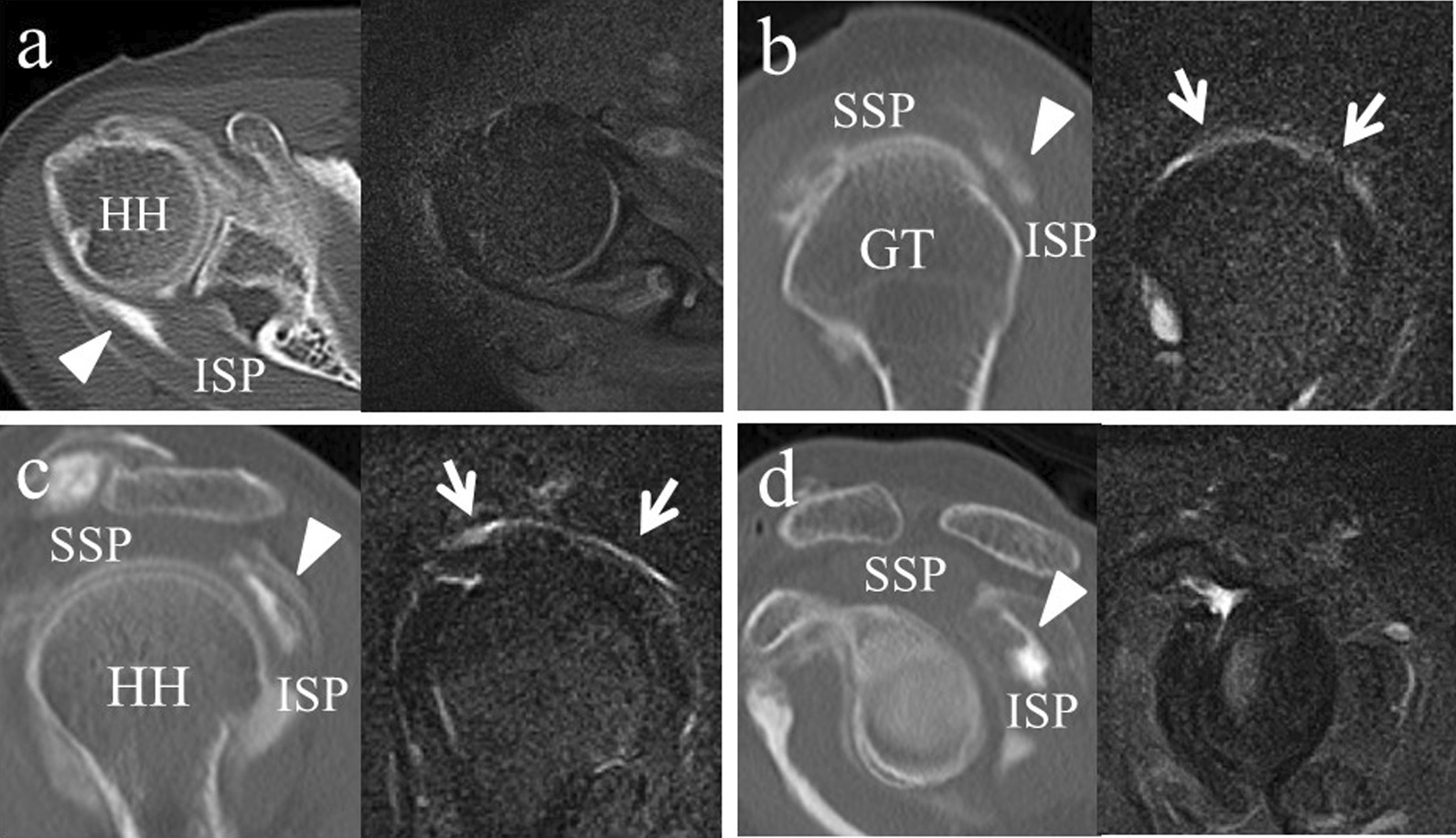
The tear is clearly visualized by Computed tomography bursography, although on fat-suppressed T2-weighted Magnetic resonance imaging, there appears to be increased signaling in the bursa, suggesting bursitis. On Computed tomography bursography, (**a**) axial image shows longitudinal pooling of contrast medium in the infraspinatus (arrowhead). **b** On Computed tomography bursography, the oblique sagittal image shows that the tear communicates with the subacromial bursa near the insertion of the infraspinatus tendon (arrowhead) and (**c**, **d**) within the infraspinatus tendon and muscle (arrowhead). Magnetic resonance imaging shows areas of increased signal in the bursa (arrows). *HH* head of the humerus, *GT* greater tubercle, *SSP* supraspinatus, *ISP* infraspinatus

A 71-year-old Asian woman presented with 3-year history of shoulder pain. She reported no history of trauma. Slight tenderness over the greater tubercle was found on physical examination, and Neer’s test and Hawkins–Kennedy test elicited pain. Plain radiographs showed no abnormal findings, and there was no apparent subacromial spur (Fig. 9). In addition, a T2-weighted MRI with a field strength of 1.5 T demonstrated a normal rotator cuff. She was diagnosed with shoulder impingement syndrome and managed conservatively for 10 months; however, her shoulder pain was refractory to conservative treatment. At 10 months, an arthrogram showed no abnormal findings and did not relieve her shoulder pain. Therefore, bursography was performed. Bursography showed pooling of the contrast medium in a tendon area of the rotator cuff (Fig. 10), and the shoulder pain was relieved. A longitudinal infraspinatus tear (Fig. 11a–d) was visualized on CT bursography. Although a fat-suppressed T2-weighted MRI (field strength, 1.5 T) was repeated within 1 month following the CT, the findings were suggestive of subacromial bursitis (Fig. 11a–d). We suspected a bursal-side infraspinatus tear; however, the patient declined surgical treatment. Her shoulder pain persisted at the 2-year follow-up.

### Technique for subacromial bursography

The patient is placed in supine position on the fluoroscopy table. Shoulder arthrography is performed first using 7–8 ml of contrast mixed with lidocaine. This contrast medium contains 10 ml 60% meglumine sodium amidotrizoate and 10 ml 2% lidocaine. If no leakage is observed on arthrography and it provides no pain relief, then subacromial bursography is performed.

The injection site for subacromial bursography is located one fingerbreadth distal to the anterolateral corner of the acromion. A 22-gauge, 32- or 70-mm needle is inserted until the tip of the needle contacts the undersurface of the acromion process [6]. When pressure is felt on the plunger of the syringe, the syringe and needle are withdrawn slowly until a sudden drop in pressure is noted, signifying that the needle tip is inside the subacromial bursa [6, 7]. Next, 3–4 ml of the same contrast mixture is injected into the bursa. Anteroposterior radiographs of the shoulder in internal, external, and neutral rotation are made. Whether the shoulder pain is relieved or not must be recorded.

Subacromial bursography was performed in each of the presented cases by a single orthopedist (Y.O.) with 3 years of experience in shoulder arthrography and bursography.

## Discussion and conclusions

The cases presented demonstrate that an isolated PTRCT can occur on the bursal-side tendon of the infraspinatus. Regarding the epidemiology of rotator cuff tears, a cadaver study of 249 supraspinatus tendons revealed that 13% had PTRCTs; of these, 55% were intratendinous tears, 27% were articular-side tears, and 18% were bursal-side tears [3, 8]. In addition, clinical studies have also shown that articular-side PTRCTs are more common than bursal-side tears [9, 10]. An arthroscopic examination of 41 partial-thickness supraspinatus tears showed bursal-side tears in 20% and articular-side tears in 80% [10]. From these reports in the literature, isolated bursal-side PTRCT of the infraspinatus is unusual.

The bursal-side infraspinatus tear may be identified as a longitudinal lesion by CT bursography due to the morphological characteristics of the infraspinatus. The infraspinatus has a long tendinous portion in the superior half of the muscle, which curves anteriorly and extends to the anterolateral area of the highest impression of the greater tuberosity [11]. Kato *et al.* [12] reported that the infraspinatus is composed of a transverse part and an oblique part in accordance with muscle fiber direction. Both parts have partially independent morphology, and the transverse part adjoins the posterior surface of the main tendinous portion of the oblique part as a thin tendinous membrane that may be fragile [12]. Therefore, longitudinal rotator cuff tears may occur when the infraspinatus is torn from the adjoining area of the tendon to the gap between the transverse and oblique muscles. This area may also become more fragile due to tendon degeneration, especially in older patients, as seen in our cases. To our knowledge, the present report is the first to document these unusual rotator cuff tears.

MRI is commonly accepted as one of the best noninvasive procedures for evaluating full-thickness rotator cuff tears and has a sensitivity and specificity of >90% [13–15]. However, the sensitivity of MRI for PTRCTs is often lower than that for full-thickness tears. A meta-analysis of 29 articles regarding the detection of rotator cuff tears demonstrated that MRI has an overall sensitivity of 67% and specificity of 94% for PTRCTs [14]. Therefore, fat suppression in MRI has been used to increase the detection of PTRCTs. Singson *et al.* [16] compared T2-weighted MRI findings with and without fat suppression in the detection of PTRCTs and found that the sensitivity of fat-suppressed MRI was 92%, versus 67% without fat suppression. Another study on the detection of PTRCTs with fat-suppressed MRI also showed a high sensitivity of 82% [17]. However, Xiao *et al.* [18] reported that fat-suppressed MRI for bursal-sided PTRCTs had a lower sensitivity of 74.3%. Hence, even with fat-suppressed MRI, it may be difficult to diagnose a bursal-side PTRCT correctly.

Shoulder arthrography is also an accepted imaging technique for evaluating rotator cuff tears. CT arthrography has a high sensitivity and specificity for the diagnosis of articular-side PTRCTs and full-thickness tears but cannot detect bursal-side PTRCTs [19, 20]. In comparison, subacromial bursography is useful for identifying bursal-side PTRCTs and full-thickness tears [21–23]. Schneider *et al.* [23] examined 17 patients with a bursal-side PTRCT and found that these were detected by MRI and ultrasonography with a sensitivity of 64% and 41%, respectively, while bursography had a higher sensitivity of 82%.

Three methods of bursography combined with other imaging examinations have been reported. First, CT bursography can visualize superficial rotator cuff lesions [24, 25]. Fermand *et al.* [25] combined bursography with CT arthrography to examine 33 patients with shoulder pain. They found that 15 of these patients had radiological abnormalities of the deep surface of the subacromial bursa, including a thumbprint-like notch in the rotator cuff muscle, serrated unevenness near the lateral edge of the rotator cuff muscle, a linear fissure in the rotator cuff tendon, unevenness of the superficial cuff surface with loose bodies in the bursa, partial avulsion of the rotator cuff tendon, and an ulcer on the superficial cuff surface. Second, ultrasonographic bursography has also been reported and noted to be more informative for rotator cuff tears than plain ultrasonography [26, 27]. Cheng *et al.* [26] used percutaneous ultrasound-guided subacromial bursography to examine 63 shoulders and found that ultrasonographic bursography correctly identified all full-thickness tears but missed one in five patients who were misdiagnosed with a bursal-side PTRCT by plain ultrasonography. Finally, MR bursography is also a possible imaging technique. However, whether it is reliable for detecting bursal-side PTRCTs remains controversial owing to the difficulty of injecting gadopentetate dimeglumine, which is not a radiocontrast agent, accurately into the subacromial bursa [28].

There are several limitations to this report to consider. First, subacromial bursography requires injection of contrast medium and therefore is invasive. Furthermore, CT exposes the patient to radiation. Second, the infraspinatus degenerative tear could be an incidental finding, which may not be the source of the patient’s symptoms and might not need isolated repair or debridement in the patients. Third, we did not demonstrate the accuracy of CT bursography in the diagnosis of bursal-side PTRCTs. However, the cases presented do provide a basis for further studies regarding bursography and its usefulness and accuracy, especially in patients with ongoing pain but normal findings on standard imaging.

In conclusion, the patients in the three cases presented had isolated bursal-side infraspinatus tears, and CT bursography was useful for identifying them. Therefore, patients with shoulder pain refractory to conservative management but normal MRI findings should be evaluated with CT bursography. Further studies are required to evaluate and compare the diagnostic accuracy of bursography combined with CT with ultrasonography and MRI for bursal-side PTRCTs.

## Data Availability

Obtained.

## Abbreviations

MRI: Magnetic resonance imaging
CT: Computed tomography
PTRCT: Partial-thickness rotator cuff tear
